# The Influence of Landscape Heterogeneity - Ground Beetles (Coleoptera: Carabidae) in Fthiotida, Central Greece

**DOI:** 10.3897/BDJ.2.e1082

**Published:** 2014-04-11

**Authors:** Anna Nicola Chapman

**Affiliations:** † National and Kapodistrian University of Athens, Athens, Greece

**Keywords:** Carabidae, Coleoptera, agriculture, farmland, cotton, maize, olives, wheat, cultivation, crops, heterogeneity, landscape, Greece

## Abstract

Pitfall traps were used to sample Carabidae in agricultural land of the Spercheios valley, Fthiotida, Central Greece. Four pairs of cultivated fields were sampled. One field of each pair was located in a heterogeneous area and the other in a more homogeneous area. Heterogeneous areas were composed of small fields. They had high percentages of non-cropped habitats and a high diversity of land use types. Homogeneous areas were composed of larger fields. They had lower percentages of non-cropped habitats and a lower diversity of land use types. One pair of fields had been planted with cotton, one with maize, one with olives and one with wheat. Altogether 28 carabid species were recorded. This paper describes the study areas, the sampling methods used and presents the data collected during the study. Neither heterogeneous nor homogeneous areas had consistently higher abundance levels, activity density levels, species richness levels or diversity levels. However, significant differences were seen in some of the comparisons between heterogeneous and homogeneous areas.

## Introduction

The level of heterogeneity in agricultural landscapes can influence farmland wildlife (Benton et al. 2003; Tscharntke et al. 2005; Fahrig et al. 2011). Heterogeneous farmland, forming a mosaic of different land use types, has been seen to benefit invertebrate groups such as the Aranea (Sunderland and Samu 2000), the Lepidoptera (Weibull et al. 2000) and the Carabidae (Östman et al. 2001), in terms of their abundance, diversity and condition.

Locally, soil moisture and soil type have a large impact on carabid distributions (Meissner 1984; Thiele 1977). However, some aspects of landscape heterogeneity will also influence the Carabidae. These aspects are field size (Kromp 1999), the presence of non-cropped habitat (Pollard 1968; Sotherton 1985) and land use diversity (Östman et al. 2001).

Small fields are thought to be easier for carabids to recolonize after disturbance, due to the shorter dispersal distances involved. Additionally, landscapes with small fields are likely to have high levels of land use diversity, which will create refuges for carabids in times of disturbance. This is because cultivation practices take place at different times in different crops, so a diverse landscape will always have some undisturbed habitat, while cultivation practices take place elsewhere (Kromp 1999).

Another aspect of landscape heterogeneity that is important for carabids, is the presence of non-cropped habitat. This may take the form of grassy field margins, hedgerows and areas of semi-natural habitat, such as fallow, woodland and wasteland. Although carabids live in the crops during the vegetation season, they are known to use field margins to hibernate in (Pollard 1968; Sotherton 1985). Where this occurs, carabids are less vulnerable to ploughing, harrowing and insecticide application, which usually take place in the autumn and winter (Holland and Luff 2000). In addition to this, field margins are known to be important as breeding sites for many carabid species (Desender and Turin 1989).

In Greece, agriculture is often extensive and small in scale, with large-scale, intensive farming occurring only on the flatter and more fertile land. This means that the country has relatively high levels of habitat richness (Eurostat 2012). Although this situation may be beneficial to wildlife, economies of scale mean that such areas are expensive to farm and have, for a long time, been prone to land abandonment and agricultural intensification (Kasimis et al. 2003; Louloudis and Maraveyas 1997). Today, these problems have been compounded with the effects of the economic crisis, meaning that small-scale farmers and the landscapes they have helped to create are now under even greater pressure (Eurostat 2012). So far, little work has been conducted on the carabid communities of farmland in Central Greece, in spite of the benefits associated with such diverse agricultural landscapes.

This study aims to identify closely matched and situated areas of heterogeneous and homogeneous farmland. These areas will then be compared using landscape analysis, so that the different aspects of their heterogeneity can be examined. Finally, matched fields of the same crop types within the heterogeneous and homogeneous areas will be compared, to see how they differ in terms of their carabid abundance, activity density, species richness and diversity. In this way, it may be determined whether high levels of landscape heterogeneity benefit Carabidae at a local level, within individual fields.

## Materials and methods

### Study Areas and Sampled Fields

The study was conducted on agricultural land in the Spercheios valley, Fthiotida, Central Greece. Four pairs of fields were sampled for Carabidae between May and October in 2007. One pair of the sampled fields had been planted with cotton, one with maize, one with olives and one with wheat.

One field of each pair was located in a heterogeneous area, which had small field sizes, a large amount of non-cropped habitat and a high level of land use diversity. The other field of each pair was located in a more homogeneous area, which had larger field sizes, less non-cropped habitat and a lower level of land use diversity (Suppl. material 2).

The heterogeneous and homogeneous areas were matched, in order to be as similar as possible regarding all factors apart from their heterogeneity. They were matched according to their mean elevation, their distances to villages and roads, as well as to large expanses of woodlandand wasteland.

**Figure 1.**
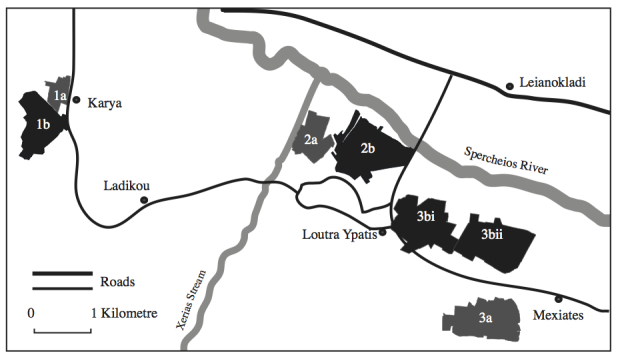
Map showing the relative positions of each of the heterogeneous (a) and homogeneous (b) areas.

Fig. 1 shows the relative positions of the different study areas. Here it can be seen that the sizes of the study areas varied and their shapes were irregular. This was because their outlines delineated the places that were considered most heterogeneous or homogeneous when compared to each other and to the surrounding countryside. When the landscape analysis was performed; however, comparisons took place between equally proportioned areas.

The sampled fields chosen from within the heterogeneous and homogeneous areas were also matched regarding their crop type, soil type, previous crop type, agrochemical treatment, harvesting time, elevation and whether or not they were irrigated. The data used to match the fields, along with the size and location of each field, are provided in Table 1.

**Figure 2.**
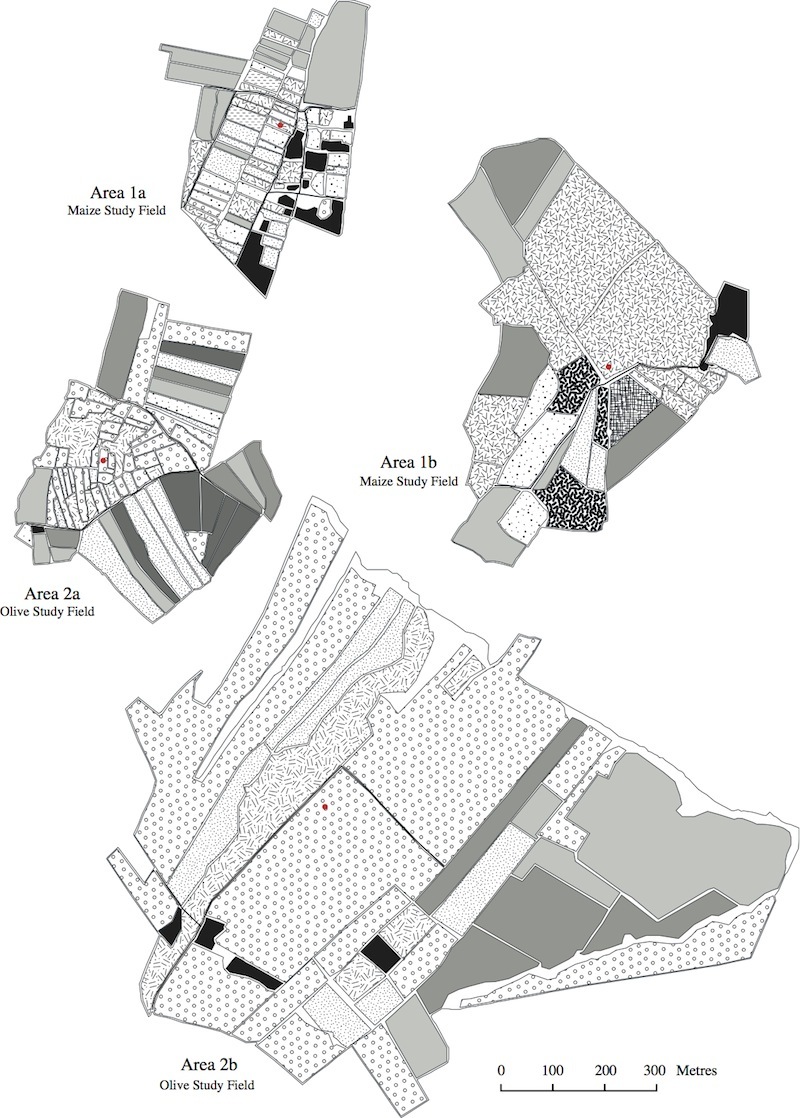
Land use maps of areas 1a, 1b, 2a and 2b. The sampled maize and olive fields within these areas are marked with red circle.

**Figure 3.**
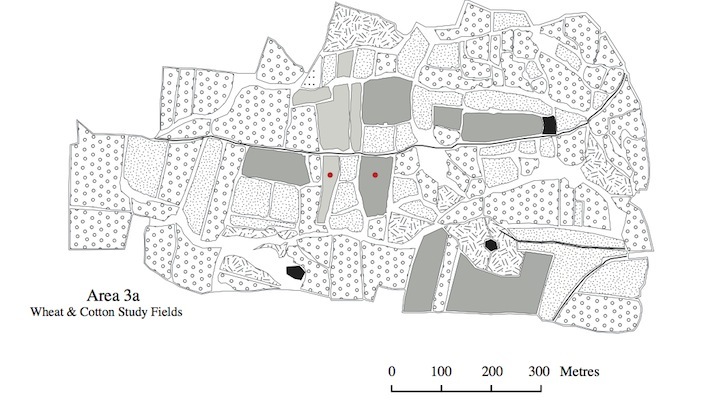
Land use map of area 3a. The sampled wheat and cotton fields within this area are marked with red circle.

**Figure 4.**
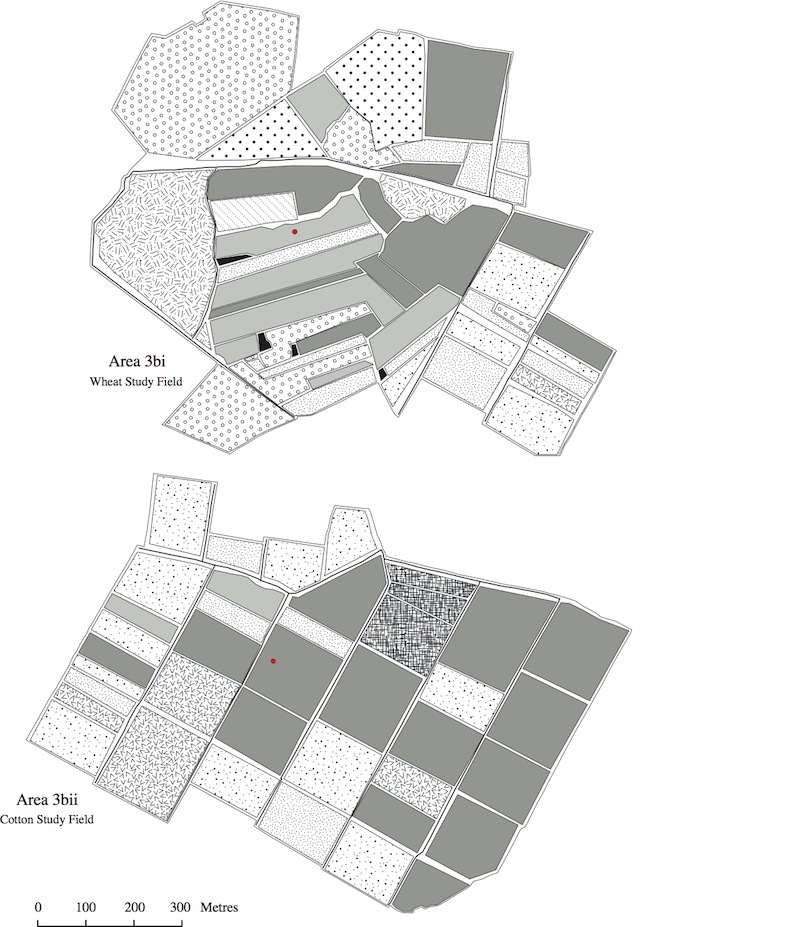
Land use maps of areas 3bi and 3bii. The sampled wheat and cotton fields within these areas are marked with red circle.

**Figure 5.**
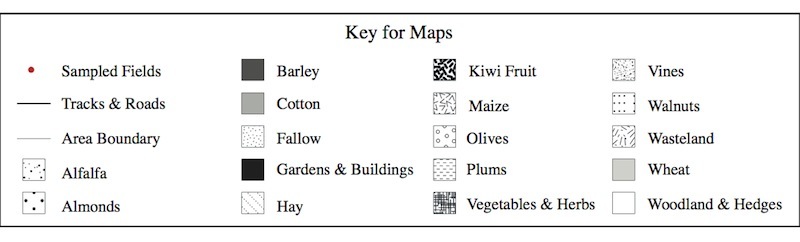
Key for the land use maps in Figs 2, 3, 4.

The land use maps in Figs 2, 3, 4 show the positions of the individual fields within the study areas. Each land use type is represented by a polygon of a different pattern and the sampled fields are marked with red circles. These maps were made using satellite photographs, taken from an eye altitude of 1.5 km. As homogeneous area 3b was very large, the map of this area was divided in two, so that two maps were made, one of area 3bi, containing the sampled wheat field and one of area 3bii, containing the sampled cotton field. The key to these maps is shown in Fig. 5.

The program FRAGSTATS (McGarigal et al. 2002) was used to conduct landscape analysis on each of the study areas, using the data presented in Figs 2, 3, 4. This resulted in the calculation of six landscape metrics (Suppl. material 2). The data for the landscape analysis were obtained by placing equal sized rectangles over the land use maps as a separate layer. The map data within these rectangles were then converted into numerical data and input into FRAGSTATS. For the metrics "land use diversity" and "land use richness" the data from 10 ha rectangles were used. This was because the results of these metrics would vary depending on the percentage of background landscape included in the analysis. For the other metrics; however, where this was not the case, 50 ha rectangles were used, meaning that all of the collected data could be analyzed.

The metric "land use aggregation" which concerns land use configuration, is dimensionless, so therefore lacks units. The same is true for the metric "land use similarity", which indicates how similar the sampled fields were, regarding land use, to other fields in the surrounding areas (McGarigal et al. 2002).

### Sampling Procedure

The locations of the pitfall traps within the sampled fields are listed as geographic coordinates in Table 1. These coordinates represent the mid points of the lines of traps. Most fields were large enough to accommodate a single line of 10 traps, situated 10 m apart, 5 m from one of the field margins. However, the olive grove and the maize field in the heterogeneous areas were too small to accommodate a single line of 10 traps. So in these fields two rows of 5 traps were used, positioned on opposite sides of the fields, 10 m apart and 5 m from each of the field margins.

Not all of the pairs of fields were sampled during every 15 day sampling period. This was because access to the fields depended on the cultivation practices taking place at the time. Irrigation, spraying, harvesting, ploughing, pruning and fertilizer application would all prevent access to the fields and the setting of traps. Initially, a pair of alfalfa fields was also sampled, but the frequency of harvesting meant that most of these traps were destroyed before they could be collected. So sampling in these fields was not continued.

If one field of a pair was inaccessible for a given 15 day period, then the other field of that pair was not sampled either. This insured that comparisons between heterogeneous and homogeneous areas were fair, as the same amount of data was obtained in both fields, at the same time of year. In all, 440 traps were set and 391 were recovered successfully. Successful traps were those that were not flooded by irrigation, destroyed by other farming practices or dug up by animals. The dates covered by the 15 day sampling periods, as well as the numbers of traps set and successfully recovered are shown in Table 2.

From the 391 successful traps, 320 (40 traps from each field) were chosen for use in the data analysis (Table 2). The number 40 was chosen because the least sampled field, "cotton a" provided a total of 42 successful traps. To begin with, two traps, chosen at random, were removed from the "cotton a" data set. Then 40 equivalent traps were chosen from the "cotton b" data set. These traps were taken from the same sampling periods as those in the "cotton a" data set. This procedure was repeated for all of the field pairs. It meant that the comparisons between fields in heterogeneous and homogeneous areas were always fair, as they were made using data from the same number of traps, taken during the same time of year.

The traps themselves were made out of 250 ml plastic cups. These had a depth of 10 cm and a rim diameter of 7.3 cm. They were part filled with ethylene glycol, and covered with wooden lids to prevent flooding and the capture of larger, non-target species. This left a gap of 2 cm between the trap rims and their lids.

The Carabidae were identified to species level using Arndt et al. (2011) and Trautner and Geigenmüller (1987). Then a subsample of the specimens, containing all of the sampled species was checked by a taxonomist from the University of Athens. A small number of specimens were also sent to the Natural History Museum in London for verification (Acknowledgements). All specimens were stored in alcohol and are now kept in the private collection of the author.

### Data Analysis

For each field, the data from 40 traps were combined, then the number of carabid species found in each field were recorded, along with their relative abundance (n). The annual activity density (ADa) (Brandmayr et al. 2005) was also calculated for each species, using the following equation:

ADa = n_tot_ / US

*Where*:

*n_tot_* = the number of individuals sampled during the season

US = sum of us

us = trap * (gg/10)

*trap* = number of traps in each field

*gg* = the number of days the traps were set for.

The diversity of carabid species was calculated for each field using the Simpson's Diversity Index (D), which is presented here as the complement (1-D).

Carabid abundance and species richness were also calculated for each trap used in the data analysis. The resulting 40-trap data sets were tested using the Anderson-Darling test. This showed that the data sets were rarely normally distributed, even after transformation, meaning that nonparametric statistics were used for significance testing. Mann-Whitney U tests were used to determine the significance of differences between heterogeneous and homogeneous areas.

To compare between the different crop types, carabid abundance and species richness were again calculated for each trap. Then the data from both fields of each crop type were combined, resulting in four, 80-trap data sets, one for each crop type. Kruskal-Wallis tests were then used to determined the significance of differences between the cotton, maize, olive and wheat cultivations.

### Results

Suppl. material 1 shows the abundance (n) and the annual activity density (ADa) of each of the carabid species sampled in the different fields. Table 3 summarizes these data and also presents the carabid species richness and diversity levels for all of the sampled fields. Suppl. material 2 presents the results of the six landscape metrics calculated for each of the study areas.

## Checklist

### Karya, Loutra Ipati, Mexiates

#### Locality

continent: Europe; country: Greece; stateProvince: Central Greece; county: Fthiotida; locality: 11 km W of Lamia. Area surrounding Karya, Loutra Ipatis and Mexiates; minimumElevationInMeters: 35 m; maximumElevationInMeters: 105 m; verbatimLatitude: 38°54'32.57"N; verbatimLongitude: 22°15'37.61"E

#### Description

Sampling took place in cotton, maize, olive, and wheat fields. It was conducted by Anna Chapman (National and Kapodistrian University of Athens) and took place between the 5^th^ of May and the 23^rd^ of October 2007. All samples were preserved in alcohol and are now kept in the author's private collection.

#### Acinopus (Acinopus) picipes

(Olivier, 1795)

##### Distribution

Western Europe to Near East and Iran (Arndt et al. 2011).

##### Notes

It digs burrows under stones and is mostly phytophagous (Trautner and Geigenmüller 1987). In this study it was found rarely (n = 2) in the wheat field of the homogeneous area.

#### Amara (Amara) aenea

(De Geer, 1774)

##### Distribution

From Macaronesia across Europe and the Mediterranean Region to Western Siberia (http://www.faunaeur.org/full_results.php?id=382134).

##### Notes

Xerophilous species, mainly inhabiting grassland, gardens, dunes and wasteland (Luff 2007). In agricultural land it is found in arable cultivations, pastureland, clover and alfalfa fields (Thiele 1977), where it prefers autumn planted crops (Holland and Luff 2000). In this study it was rare. It was found in the maize field in the heterogeneous area (n = 2) and in the olive grove in the homogeneous area (n = 2).

#### Amara (Amara) similata

(Gyllenhal, 1810)

##### Distribution

Near transpalaearctic (Arndt et al. 2011).

##### Notes

It prefers damp areas, riverbanks and water meadows (Anderson et al. 2000), but it will also inhabit arable fields (Popovic and Štrbac 2010), where it favours autumn planted crops (Holland and Luff 2000). It is a polyphagous species. The adults feed mainly on seeds, which they find by climbing into the vegetation. However, they can also prey on invertebrates (Thiele 1977). This species was found in small numbers in the maize field in the heterogeneous area (n = 5), the olive grove in the homogeneous area (n = 5) and the wheat field in the homogeneous area (n = 2).

#### Brachinus (Brachynidius) explodens

(Duftschmid, 1812)

##### Distribution

Europe to Central Asia. It is a very common species in Greece (Arndt et al. 2011).

##### Notes

Prefers dry grassland and agricultural land, where it may be found in arable cultivations and alfalfa. It is one of the most common species of Carabidae to be found in cultivated areas in Eastern Europe (Thiele 1977). It is a mesoxerophilous and zoophagous species (Varvara and Apostol 2008). In this study, it was found in small numbers in the maize field in the heterogeneous area (n = 4), the olive grove in the homogeneous area (n = 2) and the wheat field in the heterogeneous area (n = 1).

#### Calathus (Bedelinus) circumseptus

(Germar, 1824)

##### Distribution

Mediterranean Europe and parts of North Africa (Torjbio 2006). It is rare in Greece (Arndt et al. 2011).

##### Notes

This species was rare in this study and was only found in the wheat field in the homogeneous area (n = 1).

#### Calathus (Calathus) korax

(Reitter, 1889)

##### Distribution

Endemic to Greece, but widespread within the country (Arndt et al. 2011).

##### Notes

This species was found in the cotton field in the heterogeneous area (n = 1), the maize field in the heterogeneous area (n = 51), the olive grove in the homogeneous area (n = 4), the wheat field in the heterogeneous area (n = 1) and the wheat field in the homogeneous area (n = 5).

#### Calathus (Neocalathus) melanocephalus

(Linnaeus, 1758)

##### Distribution

Throughout Europe, to Western Asia and North Africa (Arndt et al. 2011).

##### Notes

In agricultural areas, it is often found on arable land, pastureland and alfalfa. It is usually absent in fields with abundant weed cover (Holland and Luff 2000). It likes open areas and is an autumn breeder (Thiele 1977). In this study, it was found in the maize field in the homogeneous area (n = 1), the olive grove in the homogeneous area (n = 3) and the wheat field in the homogeneous area (n = 1).

#### Carabus (Oreocarabus) preslii

(Dejean & Boisduval, 1830)

##### Distribution

Greece, Italy and the Balkans (http://www.faunaeur.org/full_results.php?id=386874).

##### Notes

In this study, this species was only found rarely (n = 3) in the olive grove in the homogeneous area.

#### Carabus (Pachystus) graecus

(Dejean, 1826)

##### Distribution

Greece, Turkey, the Balkans and the Middle East (http://www.faunaeur.org/full_results.php?id=386941). Within Greece it is found on the mainland and Peloponnisos (Arndt et al. 2011).

##### Notes

In this study, it was found in the olive grove in the heterogeneous area (n = 3), the olive grove in the homogeneous area (n = 7), the wheat field in the heterogeneous area (n = 3) and the wheat field in the homogeneous area (n = 12).

#### Carterus (Carterus) rufipes

(Chaudoir, 1843)

##### Distribution

Eastern European, the Mediterranean region, the Balkan Peninsula, the Caucasus, Asia Minor and the Near East (Arndt et al. 2011).

##### Notes

It is a phytophagous and xerophilous species (Pavlícek et al. 2005). In this study, it was found in the maize field in the homogeneous area (n = 2), the olive grove in the heterogeneous area (n = 1) and the wheat field in the heterogeneous area (n = 5).

#### Carterus (Carterus) rotundicollis

(Rambur, 1837)

##### Distribution

The Western Mediterranean and the Balkan Peninsula (Arndt et al. 2011).

##### Notes

It prefers open countryside (Brandmayr et al. 2006). In this study, it was rare (n = 1) and was found only in the wheat field in the homogeneous area.

#### Cylindera germanica

(Linnaeus, 1758)

##### Distribution

Europe and large parts of Asia (Arndt et al. 2011).

##### Notes

Found on loamy soil, often on flood plains (Arndt et al. 2011). It is a spring breeder, mesophilous, zoophagous and prefers to live in grassland and agricultural areas (Varvara and Apostol 2008). In this study, it was found in the cotton field in the homogeneous area (n = 1), the maize field in the heterogeneous area (n = 5) and the maize field in the homogeneous area (n = 93).

#### Dixus obscurus

(Dejean, 1825)

##### Distribution

The Balkans, Cyprus, Asia Minor, Iran, Iraq, the Caucasus and Southern Russia (Arndt et al. 2011).

##### Notes

It was found in the olive grove in the heterogeneous area (n = 14) and the wheat field in the heterogeneous area (n = 3).

#### Harpalus (Harpalus) atratus

(Latreille, 1804)

##### Distribution

Europe (except the north) and the Balkans, where it prefers foothills to alpine regions (Arndt et al. 2011).

##### Notes

It is a mesoxerophilous and polyphagous species, which prefers to live in forested areas (Varvara and Apostol 2008). It feeds on a mixture of seeds from agricultural crops, weeds and shrub species (Lundgren 2009). In this study, it was only found in the wheat field in the homogeneous area (n = 1).

#### Harpalus (Harpalus) dimidiatus

(Rossi, 1790)

##### Distribution

Western Europe to the Caucasus and the Middle East (Arndt et al. 2011).

##### Notes

A species of dry grassland, which prefers moderate temperatures and humidity levels (Thiele 1977). It is polyphagous and has been seen to consume the seeds of *Daucus* *sp.* (Lundgren 2009). In this study, it was found only in the olive grove in the heterogeneous area (n = 4).

#### Harpalus (Harpalus) smaragdinus

(Duftschmid, 1812)

##### Distribution

Most of Europe (except the north), Asia Minor, east to Western Siberia and Western China, where it prefers plains to mountains (Arndt et al. 2011).

##### Notes

It found rarely in the olive grove in the homogeneous area (n = 1) and the wheat field in the heterogeneous area (n = 1).

#### Harpalus (Pseudoophonus) rufipes

(De Geer, 1774)

##### Distribution

From the Azores, across Europe, to North Africa and Western China (Arndt et al. 2011).

##### Notes

It is polyphagous and prefers open, dry habitats and light soils. It is most often found on arable land (Luff 2007 & Thiele 1977). In this study, the species was common. It was found in the cotton field in the homogeneous area (n = 12), the maize field in the heterogeneous area (n = 622), the maize field in the homogeneous area (n = 716), the olive grove in the homogeneous area (n = 2) and the wheat field in the homogeneous area (n = 16).

#### Microlestes luctuosus

(Holdhaus, 1904)

##### Distribution

Southern Europe and Southwest Asia, widespread and common in Greece (Arndt et al. 2011).

##### Notes

It prefers warm, dry places (Cárdenas Talaverón and Piella 1985) on clayey soils (Trautner and Geigenmüller 1987). It may be found in areas of tall vegetation, those of over 20 cm in height (Fadda et al. 2008). In this study it was found in the cotton field in the heterogeneous area (n = 2), the cotton field in the homogeneous area (n = 1), the olive grove in the heterogeneous area (n = 57), the olive grove in the homogeneous area (n = 6), the wheat field in the heterogeneous area (n = 649) and the wheat field in the homogeneous area (n = 142).

#### Olisthopus fuscatus

(Dejean, 1828)

##### Distribution

Southern and Western Europe, as well as the Near East (http://www.faunaeur.org/full_results.php?id=379434).

##### Notes

Zoophagous (Vanbergen et al. 2010). It was only found rarely in the olive grove in the homogeneous area (n = 2).

#### Ophonus (Hesperophonus) azureus

(Fabricius, 1775)

##### Distribution

Northwestern Africa, Northern, Central and Southern Europe, the Balkans, the Caucasus, Asia Minor and Northwestern China (Arndt et al. 2011).

##### Notes

Consumes the seeds of common agricultural weed species such as *Capsella bursa-pastoris*, *Taraxacum officinale* and *Cirsium arvense* (Petit et al. 2011). In this study, it was rare (n = 1) and was only found in the olive grove in the heterogeneous area.

#### Ophonus (Ophonus) diffinis

(Dejean, 1829)

##### Distribution

From the Iberian Peninsular, through Southern and Central Europe, the Balkans, to the Near East and the Caucasus (Arndt et al. 2011).

##### Notes

It is polyphagous, taking insect prey, but is also known to feed on the fallen seeds of plants in the Apiaceae family (Lundgren 2009). In this study, it was rare (n = 1) and was only found in the wheat field in the homogeneous area.

#### Pachycarus (Mystropterus) cyaneus

(Dejean, 1825)

##### Distribution

Greece, FYROM (Former Yugoslav Republic of Macedonia) Bulgaria and Turkey (Arndt et al. 2011).

##### Notes

It lives in burrows underneath stones. A xerophilous species, preferring areas with sparse vegetation (Sienkiewicz 2008). In this study, it was found in the maize field in the heterogeneous area (n = 1), the olive grove in the heterogeneous area (n = 5) and the olive grove in the homogeneous area (n = 8).

#### Pangus scaritides

(Sturm, 1818)

##### Distribution

Southern Russia, the Caucasus, Iran, Asia Minor, the Balkans as well as Southern and Central Europe (Arndt et al. 2011).

##### Notes

This species was rare (n = 1) and was only found in the olive grove in the homogeneous area.

#### Poecilus cupreus

(Linnaeus, 1758)

##### Distribution

Europe, Asia Minor, Central Asia and Siberia (Arndt et al. 2011).

##### Notes

It may be found in woodland, arable land, meadows, pastures and alfalfa fields. It is one of the most common carabid species of agricultural land in Central Europe. It feeds on species of Arachnida, Acari, Staphylinidae, Thysanoptera, Aphidoidea, other Hemiptera species, Cantharidae, Coccinellidae, Chrysopa and Lepidoptera larvae (Thiele 1977). In this study it was found in the cotton field in the homogeneous area (n = 1), the maize field in the heterogeneous area (n = 47), the maize field in the homogeneous area (n = 2) and the olive grove in the heterogeneous area (n = 1).

#### Pterostichus (Platysma) niger

(Schaller, 1783)

##### Distribution

Europe, Turkey, Iran, the Caucasus, Central Asia, Mongolia, Siberia and the Far East (Arndt et al. 2011).

##### Notes

It is found in woodland, heathland and damp grassland (Luff 2007). It is zoophagous, thermophilous, xerophilous and an autumn breeder. It lives close to field margins and hedgerows (Thiele 1977). It is common in spring planted crops and in those where minimum tillage has been practiced (Holland and Luff 2000). In this study, it was found in the cotton field in the heterogeneous area (n = 1), the cotton field in the homogeneous area (n = 15), the maize field in the heterogeneous area (n = 153), the maize field in the homogeneous area (n = 82), the olive grove in the heterogeneous area (n = 11), the wheat field in the heterogeneous area (n = 1) and the wheat field in the homogeneous area (n = 17).

#### Tapinopterus (Tapinopterus) taborskyi

(Mařan, 1939)

##### Distribution

Endemic to Greece and only ever found on Oiti mountain (Arndt et al. 2011).

##### Notes

This species was found in the cotton field in the heterogeneous area (n = 2) and in the cotton field in the homogeneous area (n = 41). Both these areas were located close to Oiti.

#### Trechus (Trechus) quadristriatus

(Schrank, 1781)

##### Distribution

Europe, the Nearctic, the Near East and North Africa (http://www.faunaeur.org/full_results.php?id=384300). It is widespread and common in Greece (Arndt et al. 2011).

##### Notes

This species was found in the maize field in the heterogeneous area (n = 1), the olive grove in the heterogeneous area (n = 1) and in the wheat field in the homogeneous area (n = 1).

#### Zabrus (Pelor) graecus

(Dejean, 1828)

##### Distribution

Greece, Bulgaria, FYROM and the Near East. Often found in Attica and on the near islands (Arndt et al. 2011).

##### Notes

This species was found in the cotton field in the heterogeneous area (n = 3), the maize field in the heterogeneous area (n = 1), the maize field in the homogeneous area (n = 1), the olive grove in the heterogeneous area (n = 7), the olive grove in the homogeneous area (n = 1), the wheat field in the heterogeneous area (n = 17) and the wheat field in the heterogeneous area (n = 6).

## Discussion

Neither the heterogeneous nor the homogeneous areas had consistently higher abundance, activity density, species richness, or diversity levels. This suggests that the level of heterogeneity of the study areas did not have a great influence on the carabid communities of the sampled fields. Areas with small field sizes, large amounts of non-cropped habitat and high land use diversity did not appear to benefit the Carabidae.

Additionally, there did not seem to be an association between any of the landscape metrics in Suppl. material 2 and the levels of carabid abundance, activity density, richness or diversity. Even the "percentage of non-cropped habitat", which is a factor thought to be particularly influential for the Carabidae and more broadly for farmland biodiversity (Holland and Luff 2000; Desender and Turin 1989; Fahrig et al. 2011) appeared not to have a consistent influence.

The results of this study do not agree with those reviewed by Benton et al. (2003), Tscharntke et al. (2005) and Fahrig et al. (2011). However, this may be because the relationship between heterogeneity and biodiversity is thought not to be linear (Fahrig et al. 2011), in which case, this study's design may have been inappropriate.

Fahrig et al. (2011) suggest that when studying the influence of landscape heterogeneity, areas should be chosen that form a gradient in terms of their heterogeneity. Here though, a paired design was used and different crop types were sampled in the different area pairs. Fahrig et al. (2011) also recommend sampling a much larger number of areas (40-60). In this study, a small number of areas (only 6) were sampled, so replication occurred within the sampled fields, rather than at the area level. While this provided information at a local level, about the sampled fields themselves, it would not have provided information at a landscape level. It is likely therefore, that if a larger number of areas had been sampled, the overall influence of landscape heterogeneity would have been clearer.

A related issue is that too few land use types may have been sampled in this study. Heterogeneity is believed to enhance biodiversity, through different species being associated with different land use types, at different times in their lives (Dunning et al. 1992; Pollard 1968; Sotherton 1985; Desender and Turin 1989). This suggests that if more land use types had been sampled, a more consistent pattern may have been seen in the results.

Additionally, the need to compare closely situated areas, matched regarding other factors apart from their heterogeneity, meant that it was difficult to choose areas that differed greatly in all aspects of their heterogeneity. For example, although "land use diversity" was high and "land use similarity" was low in all of the heterogeneous areas, "land use richness" did not always follow this pattern. For the wheat comparison, "land use richness" was slightly higher in the homogeneous area, while for the cotton comparison "land use richness" was the same in both areas (Suppl. material 2). This implies that the heterogeneous and homogeneous areas may not have been different enough to influence the Carabidae present within them. Having said that though, highly homogeneous farmland was not present in the Spercheios Valley, so comparing widely different heterogeneity levels would have meant comparing areas from different regions, something which may have resulted in unfair comparisons.

It is also possible, as mentioned by Fahrig et al. (2011), that while appearing to be heterogeneous in satellite photographs, the heterogeneous areas in this study were not functionally heterogeneous from the point of view of the Carabidae. The different land use types in the heterogeneous areas may have actually provided similar resources for the Carabidae. Woodland and tree crops, for example, may have been functionally similar land use types, due to their low disturbance levels and the rich plant communities they supported.

Despite these issues, significant differences were seen in some of the comparisons between heterogeneous and homogeneous areas (Following Subsections). These results are interesting as they provide information about how the Carabidae within individual fields are affected by heterogeneity at the landscape level.

### Relative Abundance

For the cotton fields, carabid abundance per trap was significantly higher in the homogeneous area (U = 1178, p = 0.0003). For the maize fields though, there was not a significant difference between the heterogeneous and homogeneous area (U = 831.5, p = 0.7642). The olive groves had significantly higher carabid abundance in the heterogeneous area (U = 586, p = 0.0404). The result of this comparison was one of the few that showed a positive influence of heterogeneity. For the wheat fields though, there was not a significant difference between the heterogeneous and homogeneous area (U = 693, p = 0.3077). Finally, when the data from all of the fields in each type of area were combined, there was no significant difference between heterogeneous and homogeneous areas (U = 12721 p = 0.9283).

On the whole, crop type appeared to have a greater influence on the Carabidae than did heterogeneity. The results of the Kruskal-Wallis test show that there was a highly significant difference in carabid abundance between the different crop types (H = 34, p = <0.0001). This was probably due to the specific microclimates created by each crop type and to the differences in husbandry that each crop required (Holland and Luff 2000). For instance, the maize fields had the highest carabid abundance levels of all crop types, probably because they were irrigated throughout the growing season and because they were not treated with insecticides or herbicides. The cotton fields, on the other hand, had the lowest overall abundance levels, probably due to the treatment of these fields with the insecticide Phosalone. Differences between crop types; however, should not have affected comparisons between heterogeneity levels, as crop type and husbandry were matched between heterogeneous and homogeneous areas.

When the abundances of individual species were considered, significant differences were seen between some heterogeneous and homogeneous areas, but these were not consistent for all of the crop type comparisons. Relative abundance patterns also varied depending on the carabid species, with some species showing higher abundances in a heterogeneous area, and others in a homogeneous area. This may have been due to differences in dispersal ability, causing variation in they way individual species experienced heterogeneity (Tscharntke et al. 2005).

For the cotton comparison, *Pterostichus (Platysma) niger* was found in significantly higher numbers in the homogeneous area (U = 1041, p = 0.0209). Then when the influence of crop type was examined, significantly greater numbers of this species were seen in the maize fields (H = 136.13, p = <0.0001). This may have been largely due to the maize fields being irrigated regularly throughout the summer, which would have provided favourable conditions for this species.

For the olive comparison, *Microlestes luctuosus* was found in significantly greater numbers in the heterogeneous area (U = 592, p = 0.0455). This species is known to prefer areas of tall vegetation (Fadda et al. 2008), which it would have found to a greater degree in the heterogeneous area, due to the presence of greater percentages of non-cropped habitat (Suppl. material 2). There was also a significant difference in the abundance of *Microlestes luctuosus* between the different crop types (H = 73.6, p = <0.0001). It was trapped in the greatest numbers in the wheat fields. This may have been because these fields remained undisturbed as stubble from early June, when harvest took place, until October, when they were ploughed. Leaving stubbles intact for many months is something that has been seen to be beneficial for many groups of species, including carabids (Evans et al. 2002).

For the maize comparison, *Poecilus cupreus* was found in significantly greater numbers in the heterogeneous area (U = 521, p = 0.0074). This may have been due to a preference for areas of non-cropped habitat at certain times of life (Thiele 1977) and could indicate a requirement for high levels of heterogeneity when inhabiting agricultural land.

For the cotton comparison, *Tapinopterus (Tapinopterus) taborskyi* was found in significantly greater numbers in the homogeneous area (U = 1050, p = 0.0164). This may suggest a preference for low heterogeneity levels. However, as this species has only ever been found on Oiti mountain (Arndt et al. 2011), which is adjacent to the Spercheios Valley, this seems unlikely. Perhaps its high abundance level in the cotton field in the homogeneous area was due to there being an easy dispersal route from Oiti in that direction, possibly due to the prevailing wind direction.

For the maize comparison, *Cylindera germanica* was found in significantly higher numbers in the homogeneous area (U = 1199, p = 0.0001). This was probably because *Cylindera germanica* is accustomed to living in agricultural areas (Varvara and Apostol 2008) so a low level of heterogeneity would not be detrimental to it. There was also a significant difference seen in the abundance of this species between the different crop types (H = 17.19, p = 0.0006), with far greater numbers being trapped in the maize fields. This was probably due to the frequent irrigation required by the maize crop. This would make sense, as *Cylindera germanica* is also known to be a common inhabitant of damp areas, such as flood plains (Arndt et al. 2011).

The most common species in this study, *Harpalus (Pseudoophonus) rufipes*, did not show any significant differences between heterogeneous and homogeneous areas. This is probably due to it being a very common inhabitant of agricultural land (Luff 2007; Thiele 1977), and so being well adapted to living in all kinds of agricultural landscapes. However, significantly greater numbers of this species were seen in the maize fields (H = 136.13, p = <0.0001). Again this was probably due to the frequent irrigation of this crop type, which would have provided moisture during the dry summer period.

### Species Richness

For the cotton fields, there were significantly higher numbers of carabid species per trap in the homogeneous area (U = 1190, p = 0.0002), something that does not indicate a positive influence of landscape heterogeneity. For the maize and wheat fields, no significant differences were seen between heterogeneous and homogeneous areas (maize U = 673.5, p = 0.2263, wheat U = 759.5, p = 0.7039). For the olive groves too, there was not a significant difference between the heterogeneous and homogeneous area (U = 644.5, p = 0.1362). Additionally, when the data from all of the fields in each type of area were combined, there was no significant difference between heterogeneous and homogeneous areas (U = 13255, p = 0.5823).

The results of the Kruskal-Wallis test; however, showed that there was a highly significant difference in species numbers per trap between the different crop types (H = 90, p = <0.0001). The olive groves and the wheat fields had the highest overall richness levels of the four crop types. This may have been because these fields were organically farmed (Eyre et al. 2009; Clark 1999; Pfiffner and Niggli 1996). Another important factor may have been that the olive groves were left relatively undisturbed for most of the year, allowing the development of rich plant communities. The only cultivation practices that took place during the sampling season were pruning and organic manure application. The wheat fields were also left undisturbed for a large part of the summer. After harvest in early June, their stubbles were left intact until they were ploughed in October, which may have conferred benefits to the Carabidae living within them (Evans et al. 2002). The cotton fields; however, had the lowest species richness levels of all the crop types, something that was probably due to the treatment of these fields with the insecticide Phosalone.

### Diversity

Carabid diversity levels were not consistently higher in either the heterogeneous or the homogeneous areas. Nor was there a clear association between carabid diversity levels and the levels of any of the landscape metrics in Suppl. material 2.

Neither were diversity levels consistently higher in any one crop type. Although overall diversity levels were highest in the olives groves, the least disturbed of all of the different cultivations. The highest diversity levels for individual fields were seen in the wheat and olive cultivations in the homogeneous areas. However, the lowest diversity level was seen in the wheat field in the heterogeneous area.

Again these results suggest that the level of heterogeneity had little influence on the Carabidae in the sampled fields. Although, as previously discussed, these findings may have been due to limitations imposed by this study's design.

## Supplementary Material

Supplementary material 1 Carabidae Data Data type: Abundance, Activity Density, Species Richness
File: oo_6077.xls Anna Chapman

Supplementary material 2 Landscape Metrics Data type: Landscape Metrics
File: oo_6078.xls Anna Chapman

XML Treatment for Acinopus (Acinopus) picipes

XML Treatment for Amara (Amara) aenea

XML Treatment for Amara (Amara) similata

XML Treatment for Brachinus (Brachynidius) explodens

XML Treatment for Calathus (Bedelinus) circumseptus

XML Treatment for Calathus (Calathus) korax

XML Treatment for Calathus (Neocalathus) melanocephalus

XML Treatment for Carabus (Oreocarabus) preslii

XML Treatment for Carabus (Pachystus) graecus

XML Treatment for Carterus (Carterus) rufipes

XML Treatment for Carterus (Carterus) rotundicollis

XML Treatment for Cylindera germanica

XML Treatment for Dixus obscurus

XML Treatment for Harpalus (Harpalus) atratus

XML Treatment for Harpalus (Harpalus) dimidiatus

XML Treatment for Harpalus (Harpalus) smaragdinus

XML Treatment for Harpalus (Pseudoophonus) rufipes

XML Treatment for Microlestes luctuosus

XML Treatment for Olisthopus fuscatus

XML Treatment for Ophonus (Hesperophonus) azureus

XML Treatment for Ophonus (Ophonus) diffinis

XML Treatment for Pachycarus (Mystropterus) cyaneus

XML Treatment for Pangus scaritides

XML Treatment for Poecilus cupreus

XML Treatment for Pterostichus (Platysma) niger

XML Treatment for Tapinopterus (Tapinopterus) taborskyi

XML Treatment for Trechus (Trechus) quadristriatus

XML Treatment for Zabrus (Pelor) graecus

## Figures and Tables

**Table 1.** The size and location of each of the sampled fields. Also the data used to match fields in heterogeneous and homogeneous areas.

Sampled Field - (Study Area)	Field Size (ha)	Location - (Trap Line Coordinates)	Mean Elevation of Field (m)	Dominant Soil Type	Insecticide	Fertilizer	Previous Crop	Harvest Time	Irrigation
Cotton a - (3a)	0.72	38°52'51.84"N 22°17'50.77"E	51	Poorly sorted, very coarse sand	Phosalone	11-15-15	Cotton	Late October	Yes
Cotton b - (3bii)	2.16	38°53'35.73"N 22°17'46.68"E	36	Poorly sorted, very coarse sand	Phosalone	11-15-15	Cotton	Late October	Yes
Maize a - (1a)	0.08	38°54'57.81"N 22°12'35.62"E 38°54'58.13"N 22°12'35.83"E	97	Very coarse, silty, very coarse sand	None	10-20-10, Lime	Maize	Mid September	Yes
Maize b - (1b)	4.76	38°54'39.10"N 22°12'24.85"E	105	Very coarse, silty, very coarse sand - Very coarse, silty coarse sand.	None	23-8-6, 0.5 Zn	Maize	Mid September	Yes
Olives a - (2a)	0.14	38°54'34.25"N 22°15'38.14"E 38°54'34.31"N 22°15'37.61"E	80	Poorly sorted, very coarse sand	None	None	Olives	Late November	No
Olives b - (2b)	10.37	38°54'27.91"N 22°16'12.65"E	70	Poorly sorted, very coarse sand - Poorly sorted, medium sand	None	None	Olives	Late November	No
Wheat a - (3a)	0.36	38°52'51.67"N 22°17'44.45"E	52	Poorly sorted, medium sand	None	None	Alfalfa	Early June	No
Wheat b - (3bi)	1.81	38°53'46.91"N 22°16'57.99"E	53	Poorly sorted, coarse sand	None	None	Alfalfa	Early June	No

**Table 2.** Sampling Procedure

**Sampled Field - (Study Area)**	**15 day Sampling Period**	**Number of Traps Set**	**Number of Successful Traps**	**Number of Traps Used in the Data Analysis**
**Cotton a - (3a)**	22^nd^ May to 6^th^ June	10	10	10
	7^th^ June to 22^nd^ June	10	9	9
	9^th^ July to 24^th^ July	10	6	5
	8^th^ Sept to 23^rd^ Sept	10	10	10
	23^rd^ Sept to 8^th^ Oct	10	7	6
	Total = 5 periods of 15 days	Total = 50	Total = 42	**Total = 40**
**Cotton b - (3bii)**	22^nd^ May to 6^th^ June	10	10	10
	7^th^ June to 22^nd^ June	10	10	9
	9^th^ July to 24^th^ July	10	5	5
	8^th^ Sept to 23^rd^ Sept	10	10	10
	23^rd^ Sept to 8^th^ Oct	10	10	6
	Total = 5 periods of 15 days	Total = 50	Total = 45	**Total = 40**
**Maize a - (1a)**	7^th^ June to 22^nd^ June	10	10	10
	23^rd^ June to 8^th^ July	10	10	9
	9^th^ July to 24^th^ July	10	10	9
	9^th^ Aug to 24^th^ Aug	10	9	8
	8^th^ Sept - 23^rd^ Sept	10	4	4
	Total = 5 periods of 15 days	Total = 50	Total = 43	**Total = 40**
**Maize b - (1b)**	7^th^ June to 22^nd^ June	10	10	10
	23^rd^ June to 8^th^ July	10	10	9
	9^th^ July to 24^th^ July	10	9	9
	9^th^ Aug to 24^th^ Aug	10	9	8
	8^th^ Sept to 23^rd^ Sept	10	7	4
	Total = 5 periods of 15 days	Total = 50	Total = 45	**Total = 40**
**Olives a - (2a)**	5^th^ May to 20^th^ May	10	8	7
	22^nd^ May to 6^th^ June	10	10	9
	23^rd^ June to 8^th^ July	10	10	9
	9^th^ July to 24^th^ July	10	10	9
	25^th^ July to 9^th^ Aug	10	10	6
	8^th^ Oct to 23^rd^ Oct	10	10	0
	Total = 6 periods of 15 days	Total = 60	Total = 58	**Total = 40**
**Olives b - (2b)**	5^th^ May to 20^th^ May	10	10	7
	22^nd^ May to 6^th^ June	10	10	9
	23^rd^ June to 8^th^ July	10	10	9
	9^th^ July to 24^th^ July	10	10	9
	25^th^ July to 9^th^ Aug	10	10	6
	8^th^ Oct to 23^rd^ Oct	10	9	0
	Total = 6 periods of 15 days	Total = 60	Total = 59	**Total = 40**
**Wheat a - (3a)**	5^th^ May to 20^th^ May	10	10	10
	23^rd^ June to 8^th^ July	10	10	8
	9^th^ July to 24^th^ July	10	10	8
	9^th^ Aug to 24^th^ Aug	10	8	7
	23^rd^ Sept to 8^th^ Oct	10	9	7
	8^th^ Oct to 23^rd^ Oct	10	0	0
	Total = 6 periods of 15 days	Total = 60	Total = 47	**Total = 40**
**Wheat b - (3bi)**	5^th^ May to 20^th^ May	10	10	10
	23^rd^ June to 8^th^ July	10	8	8
	9^th^ July to 24^th^ July	10	10	8
	9^th^ Aug to 24^th^ Aug	10	7	7
	23^rd^ Sept to 8^th^ Oct	10	10	7
	8^th^ Oct to 23^rd^ Oct	10	7	0
	Total = 6 periods of 15 days	Total = 60	Total = 52	**Total = 40**

**Table 3.** The total abundance (N), total annual activity density (ADa), species richness and diversity (1-D) of Carabidae in each field.

Sampled Field - (Study Area)	Total Abundance (N)	Total Annual Activity Density (ADa)	Species Richness	Diversity (1-D)
Cotton a - (3a)	9	0.150	5	0.86
Cotton b - (3bii)	71	1.183	6	0.60
Maize a - (1a)	892	14.869	11	0.48
Maize b - (1b)	897	14.950	7	0.34
Olives a - (2a)	105	1.750	11	0.69
Olives b - (2b)	47	0.683	14	0.97
Wheat a - (3a)	681	11.350	9	0.09
Wheat b - (3bi)	208	3.467	14	0.97
